# Mechanical Properties of Full-Scale UHPC-Filled Steel Tube Composite Columns under Axial Load

**DOI:** 10.3390/ma16134860

**Published:** 2023-07-06

**Authors:** Baoquan Cheng, Weichen Wang, Junhua Li, Jianling Huang, Huihua Chen

**Affiliations:** 1 School of Civil Engineering, Central South University, Changsha 410083, China; curtis_ch@csu.edu.cn (B.C.); hjl1201@csu.edu.cn (J.H.); 2 School of Civil Engineering and Geographical Environment, Ningbo University, Ningbo 315211, China; ningbodxwwc@163.com (W.W.); lijunhua@nbu.edu.cn (J.L.)

**Keywords:** composite column, full-scale FUCFSTcs, axial load, failure mode, limit bearing capacity, ABAQUS

## Abstract

In the realm of civil engineering, ultra-high-performance concrete-filled steel tube composite columns (UCFSTCs) constitute a new type of building material and structure, exhibiting high compressive strength and commendable durability. Given their promising characteristics, the prospects of their application are highly promising and are worthy of further exploration. However, current research has primarily focused on scaled-down specimens, thereby limiting a broader understanding of UCFSTCs’ full-scale mechanical properties in real-world scenarios. This study aimed to investigate the mechanical properties of full-scale UHPC-filled steel tube composite columns (FUCFSTCs) in practical engineering applications. With the steel tube strength, steel tube thickness, concrete strength, aspect ratio, and steel tube diameter used as design parameters and the finite element software ABAQUS as the analytical tool, a total of 21 FUCFSTCs were designed and analyzed. Through a comparison with experimental curves, the rationality of both the material constitutive model and finite element model was verified, and the maximum error was 6.54%. Furthermore, this study analyzed the influence of different design parameters on FUCFSTCs’ ultimate bearing capacity, ductility coefficient, and the stress–strain relationship of their concrete. The ductility coefficient remained around 1.3, and the cross-sectional size had the greatest impact on the bearing capacity of the composite column, with a maximum increase of 145.90%. Additionally, this paper provides an in-depth analysis of FUCFSTCs’ mechanical behavior, failure mode, and stress process under an axial load. In conclusion, this research proposes an axial compression limit bearing capacity formula for FUCFSTCs via statistical regression, with a maximum error of 3.04%, meeting engineering accuracy requirements. Consequently, this study lays a strong foundation for the future application of FUCFSTCs in practical engineering.

## 1. Introduction

The evolving strength requirements of contemporary building structures have necessitated the exploration of alternatives to conventional reinforced concrete structures, which often fall short in high-rise and large-span designs. Consequently, concrete-filled steel tube composite structures (CFSTcs) have emerged as a viable solution, demonstrating exceptional bearing capacity, deformation capacity, and durability in several studies [1,2,3,4,5]. Alongside this development, advancements in concrete materials have spurred the study of ultra-high-performance concrete (UHPC), a novel concrete material with distinct properties [6,7]. However, despite its ultra-high compressive strength, UHPC exhibits brittleness when used in isolation. Hence, integrating UHPC with CFST technology has become a critical development trend to enhance its performance.

A series of notable investigations into the performance of UHPC-filled steel tube columns (UCFSTcs) under axial compression are worth highlighting. Guler et al. [8] performed axial compression mechanical tests on UCFSTcs in 2013, providing insight into the potential for increased structural ductility after the peak load by thickening the steel tube. Subsequent research by Xiong et al. [9] in 2017 encompassed 56 sets of axial compression tests on UCFSTcs and UCFDSTcs, which underscored the ability of the simultaneous use of UHPC and UTC to significantly boost the ultimate bearing capacity of composite columns. Their findings also suggest caution against the use of steel-fiber-reinforced UHPC and provide a comparative analysis with existing code calculation results. In 2018, Chen et al. [10] examined the axial compression characteristics of UCFSTcs, elucidating how steel tubes can effectively constrain concrete, thereby mitigating the degree of brittle failure of UHPC. Yan et al. [11] further explored this in 2019, investigating the axial compressive mechanical properties of square UCFSTcs and revealing the significant impact of the interaction between steel tubes and UHPC on ductility. Similarly, Semendary et al. [12] conducted a study on the bonding performance between high-strength concrete (HSC) and UHPC at different ages and with different surface treatments, providing valuable data for predicting bond and slip relationships. More recent investigations, such as those by Hoang et al. [13], Chang et al. [14], Xu et al. [15], and Wei et al. [16], have expanded our understanding of UCFSTcs under axial compression. In particular, these studies identified key factors affecting the strength and ductility of UCFSTcs, such as the effect of loading the concrete core only [13], the failure mode of the specimen [14,15], and the impact of high-strength steel tubes [15,16]. In 2021 and 2022, Li et al. [17,18] and Ji et al. [19] further extended this field of research, examining the flexural performance of high-strength double steel tube ultra-high-performance concrete composite columns (UHPCFDST) and the mechanical properties of elliptical high-strength steel tube UHPC composite columns under eccentric compression, respectively. Their findings indicate the critical role of the steel tube strength and thickness in improving the ultimate bearing capacity of composite columns.

Despite the wealth of research on UHPC composite columns with steel tubes, most studies have primarily focused on experimentally investigating reduced-scale composite columns. The behavior of full-scale UHPC composite columns with steel tubes has not been thoroughly documented. This paper seeks to fill this gap by analyzing the mechanical properties, stress mechanisms, and failure mode of full-scale UHPC-filled steel tube composite columns (FUCFSTcs) under axial load using ABAQUS finite element software. Furthermore, this paper proposes an ultimate bearing capacity formula for FUCFSTcs under axial compression based on statistical regression. This work aims to contribute a theoretical foundation for the application of FUCFSTcs in practical engineering, thereby advancing the field toward improved designs for high-rise and large-span structures.

## 2. Specific FUCFSTcs Design Parameters

**Figure 1:**
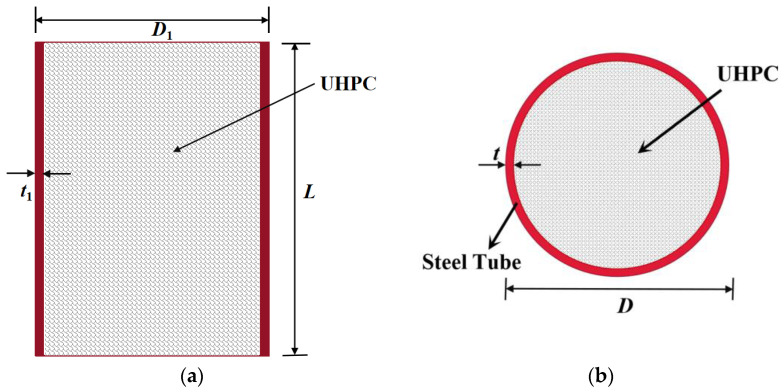
Schematic cross-section of FUCFSTcs. (**a**) Longitudinal section diagram, (**b**) Transverse section diagram.

In order to study the axial compressive mechanical properties of full-scale steel tube UHPC composite columns (FUCFSTcs), 21 FUCFSTcs were designed with the steel tube strength ***f*y**, steel tube thickness ***t***, concrete strength ***f*c**, slenderness ratio ***λ***, steel tube diameter ***D***, and composite column height ***L*** as parameters. The specific parameters of the specimen are shown in Table 1, and the sectional diagram of the composite column is shown in Figure 1. The slenderness ratio calculation formula is *λ* = *L*/*D*.

## 3. FE Model

The constitutive model of steel utilized in this article adopts a bilinear model that accounts for the plastic hardening effect [19]. Equation (1) shows the calculation expression, where the elastic modulus of steel is set at 206,000, and Poisson’s ratio is set at 0.3. In addition, the yield strain and yield strength of steel are denoted by *ε*_y_ and *f*_y_, respectively, with the assumption that *E* is 0. This approach ensures a rigorous and accurate characterization of the mechanical properties of steel in the study.

(1) $$\sigma =\left\{\begin{array}{l} E_{s}\times \varepsilon \;(\varepsilon \leq \varepsilon_{y})\\ f_{y}\;\;(\varepsilon >\varepsilon_{y}) \end{array}\right.$$

**Figure 2:**
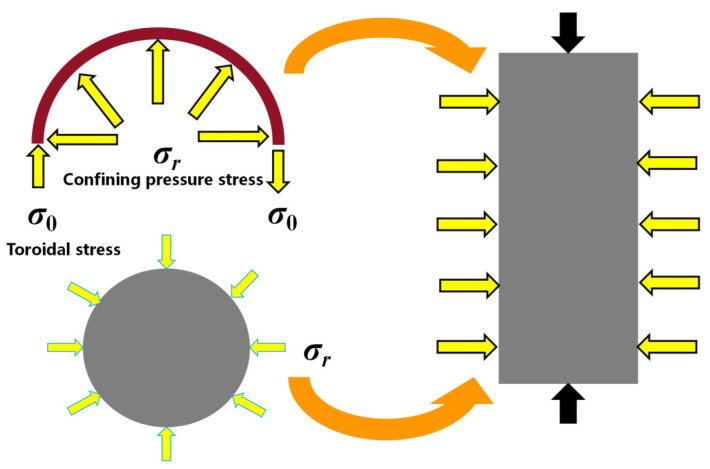
Stress state of concrete.

The stress pattern of concrete under the confinement of a steel tube is illustrated in Figure 2, revealing that under axial loads, the concrete is in a three-way stress state. Consequently, the use of a non-restrained concrete constitutive model cannot precisely capture the mechanical properties of concrete under the confinement of steel tubes. While Han et al. [20], Tao et al. [21], Teng et al. [22], and others have proposed nonlinear restrained concrete constitutive models, the model proposed by Han is only applicable to ordinary concrete, and the model proposed by Teng is only applicable to GFRP restraint. As this study focused on UHPC, it employed the nonlinear restrained concrete constitutive model applicable to ultra-high-strength concrete proposed by Tao et al., as shown in Equations (2) and (3) for compression and tension, respectively.

The stress–strain relationship of concrete under uniaxial compression is:

(2) $$\frac{\sigma }{f_{c}}=\left\{\begin{array}{c} \frac{(\mathrm{ax}+bx^{2})f_{c}}{1+\left(a-2\right)x+(b-1)x^{2}}\;\;0<x\leq 1\\ f_{r}+\left(f_{c}-f_{r}\right)exp{\left[-{\left(\frac{\varepsilon -\varepsilon_{\mathrm{cc}}}{\alpha }\right)}^{\beta }\right]}^{2}\;\;\varepsilon >\varepsilon_{\mathrm{cc}} \end{array}\right.$$

where $x=\varepsilon /\varepsilon_{c0}$, $c=\frac{E_{c}\varepsilon_{c0}}{f_{c}}$, $d=\frac{{(A-1)}^{2}}{0.55}-1$, $\varepsilon_{0}=0.00076+{\left[(0.626f_{c}-4.33)\times 10^{-7}\right]}^{0.5}$, $\varepsilon_{\mathrm{cc}}=\varepsilon_{0}\times E^{\left(k\right)}$, $k=(2.9224+0.0036f_{c}){\left(\frac{f_{b}}{f_{c}}\right)}^{0.3124+0.02f_{c}}$, and $a=0.04-\frac{0.036}{1+e^{-1.38\xi_{c}-3.49}}$. In these equations, *f*_r_ represents residual stress, *f*_c_ represents the ultimate compressive strength of concrete, β is taken as 1.2 (for circular steel tubes), and $\varepsilon_{0}$ is the peak strain corresponding to the peak stress.

In this paper, the failure energy criterion of concrete is adopted to consider the tensile softening performance:

(3) $$G_{F}=(0.049d_{\max}^{2}-0.5d_{\max}+26)\times {\left(\frac{f_{c}}{10}\right)}^{0.7}N/m$$

where *d*_max_ = 20 mm.

## 4. Establishment of Full-Scale FUCFSTcs Model

**Figure 3:**
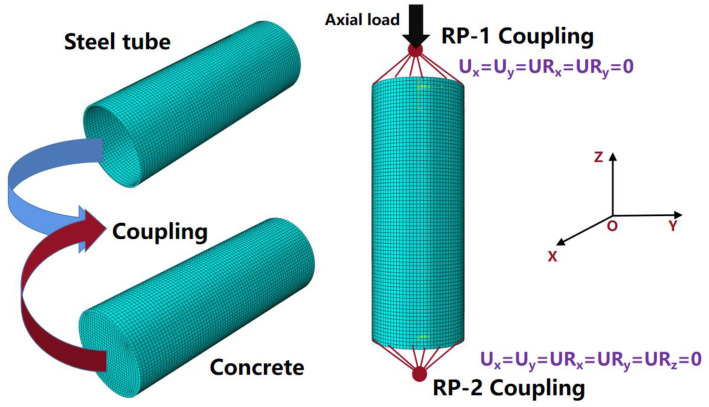
Diagram of the FUCFSTcs finite element model.

In this study, a finite element model of a full-scale EUCFDST composite column under axial load was established using the large-scale finite element analysis software ABAQUS. The FUCFST composite column was modeled using eight-node three-dimensional solid elements (C3D8R). Two reference points, RP-1 and RP-2, were defined at the top and bottom ends of the test piece and coupled with the top and bottom faces of the column. Specifically, while the displacement load was set at RP-1, the degrees of freedom at RP-1 were constrained to U_x_ = U_y_ = UR_x_ = UR_y_ = 0, and the degrees of freedom at RP-2 were constrained to U_x_ = U_y_ = U_z_ = UR_x_ = UR_y_ = UR_z_ = 0, where U_x_, U_y_, and U_z_ are the displacements of the RP point in the x-, y-, and z-directions, respectively, and UR_x_, UR_y_, and UR_z_ are the angles of rotation of the RP point in the x-, y-, and z-directions, respectively. The mesh was partitioned using hexahedral elements with a mesh size of 40 mm, as shown in Figure 3. Additionally, a normal-direction hard contact was defined between the steel tube and concrete, with a penalty function set at 0.6 in the tangential direction. These model parameters and boundary conditions were meticulously selected to ensure accurate and precise simulation results for the EUCFDST composite column.

## 5. Verification of Constrained UHPC Constitutive Model Rationality

**Figure 4:**
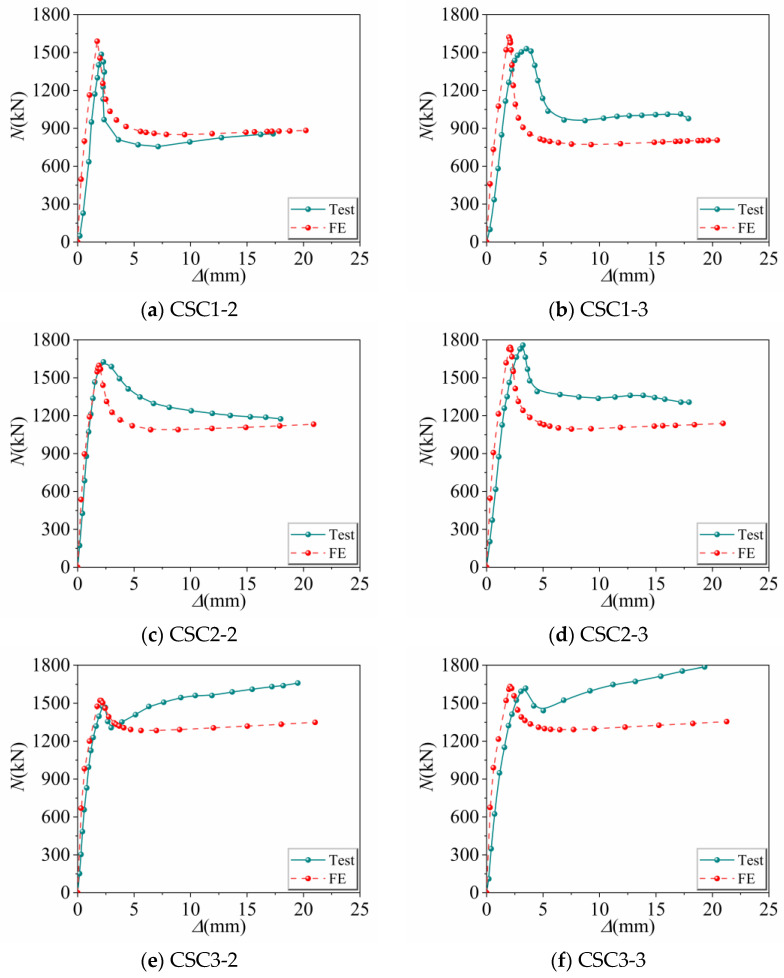

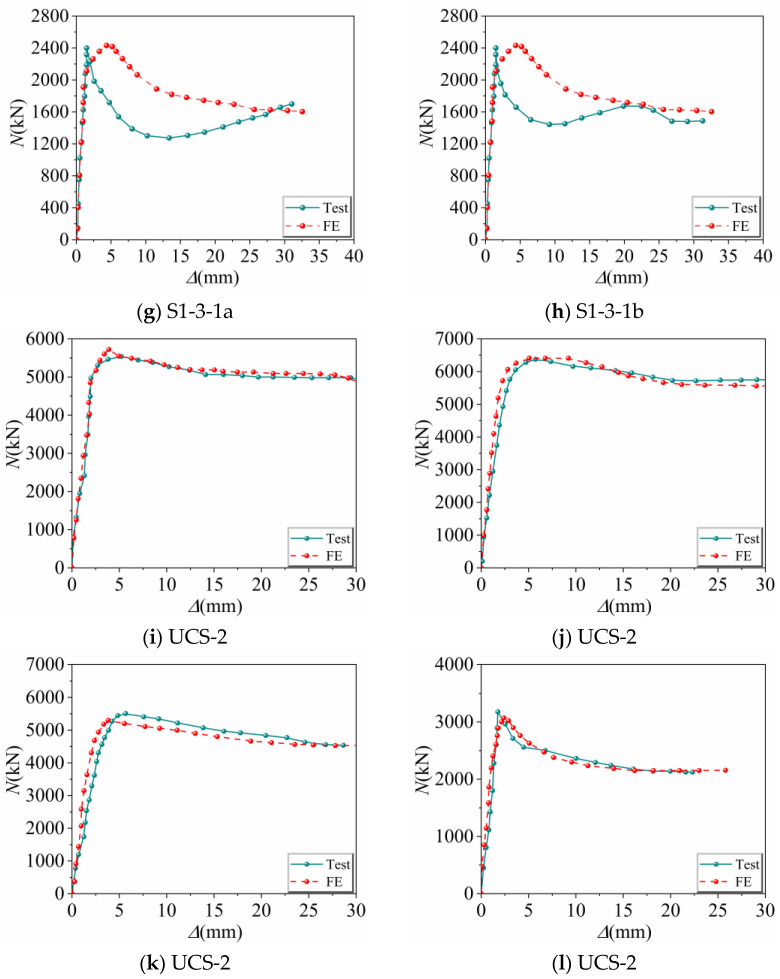
Comparison of test and finite element load–displacement curves.

**Figure 5:**
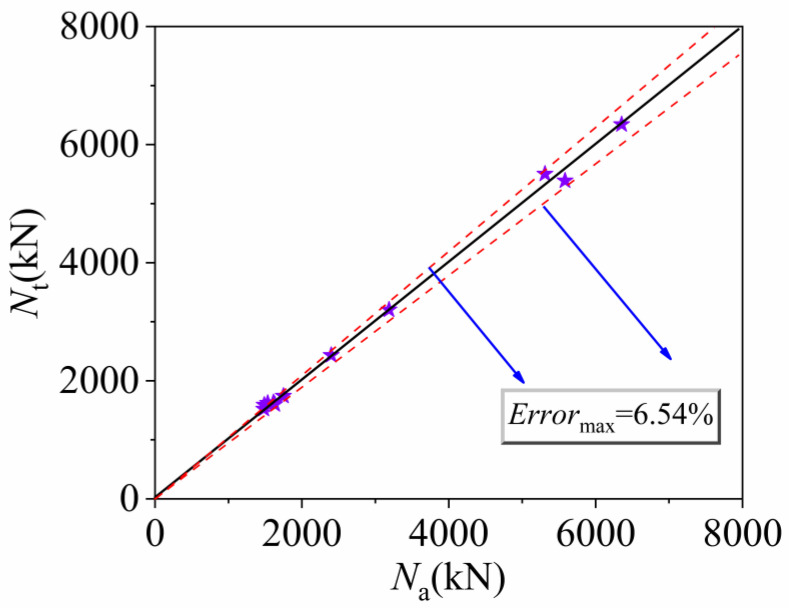
Scatter plot of experimental and FE ultimate bearing capacity errors, purple Star represents the error position of ultimate bearing capacity in simulation and testing. Note: *N*_a_ is the finite element ultimate bearing capacity value, and *N*_t_ is the experimental ultimate bearing capacity value.

To assess the validity of the constrained UHPC constitutive model, this study selected twelve groups of axial compression tests on UHPCFSTcs conducted by Chen et al. [10], Richard et al. [23], and Wei et al. [15], with their specific parameters listed in Table 2. The 12 specimens were analyzed using ABAQUS finite element software, and the resulting load–displacement curves were compared with the experimental curves. As shown in Figure 4, apart from the last four groups of specimens, the load–displacement curves of the initial six groups of specimens in the late loading phase do not exhibit complete similarity, showing a certain degree of deviation. This discrepancy can be attributed to the restraining conditions present between the steel tube and the concrete during late-stage test loading, as well as the inherent variability introduced by the finite element simulation when modeling concrete damage. The comparison revealed good agreement between the simulation and experimental curves. Moreover, the ultimate bearing capacity obtained through finite element analysis was compared with the experimental data (also presented in Table 2), and the corresponding error scatter plot is illustrated in Figure 5. The occurrence of a 6.54% error is attributed to the inability of the constitutive model used in this study to fully characterize the mechanical properties of all types of non-fiber-reinforced ultra-high-performance concrete (UHPC) mentioned in this article. Due to regional and testing method variations, not all UHPC compositions are identical. Therefore, a discrepancy of up to 10% between the simulated load–displacement curve and the experimental curve is considered acceptable in practical engineering applications.

## 6. Analysis of Load–Displacement Curves

**Figure 6:**
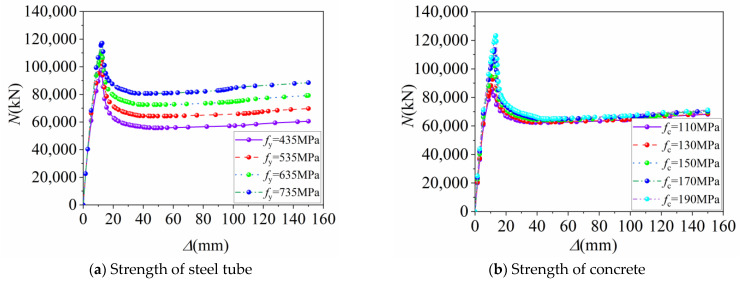

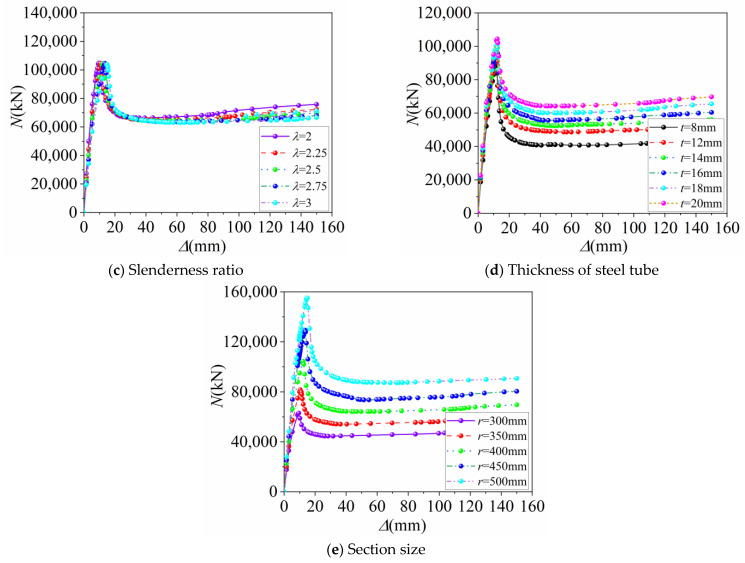
Load–displacement curves of 20 full-scale FUCFST composite columns.

Figure 6a presents the axial load–displacement curves of FUCFSTcs for various external steel tube strengths, while Table 3 shows the ultimate axial compressive load capacity, ultimate displacement, and ductility coefficient of the 20 composite columns. As the external steel tube strength increases from 435 MPa to 535 MPa, 635 MPa, and 735 MPa, the ultimate bearing capacity of the composite columns increases from 97,758.9 kN to 104,467 kN, 110,960 kN, and 117,093 kN, respectively, resulting in respective increases of 6.86%, 13.05%, and 19.78%. Correspondingly, the ultimate displacement increases from 11.96 mm to 12.23 mm, 12.45 mm, and 12.63 mm, respectively, with increases of 2.26%, 4.10%, and 5.60%. Moreover, the ductility coefficient of the composite columns increases from 1.35 to 1.39, 1.41, and 1.43, respectively, with increases of 2.96%, 4.44%, and 5.93%. These results demonstrate that as the steel tube strength gradually increases, the ultimate bearing capacity, ultimate displacement, and ductility of FUCFSTcs improve correspondingly. Notably, Figure 6a indicates that with the gradual increment in the external steel tube strength, the post-peak carrying capacity of FUCFSTcs gradually increases, and the load–displacement curve is relatively stable in the post-loading stage, indicating that this type of composite column exhibits robust post-peak stability.

From Figure 6b, it is evident that the ultimate axial compressive load capacity of the composite column gradually increases as the strength of the concrete increases. The rate of curvature reduction after reaching the peak load gradually increases, and the load-bearing capacity of the composite column in the later stages of loading shows no significant differences. Specifically, as the concrete strength increases successively from 110 MPa to 130 MPa, 150 MPa, 170 MPa, and 190 MPa, the ultimate load capacity of the composite column increases from 85,665.4 kN to 94,037.9 kN, 104,467 kN, 113,527 kN, and 123,351 kN, with increases of 9.77%, 21.95%, 32.52%, and 43.99%, respectively. However, the ultimate displacement of the composite column increases from 11.07 mm to 11.59 mm, 12.23 mm, 12.54 mm, and 13.37 mm, representing increases of 4.70%, 10.48%, 13.28%, and 20.78%, respectively, and the ductility coefficient similarly diminishes from 1.76 to 1.48, 1.39, 1.32, and 1.27, signifying decreases of 15.91%, 21.02%, 25.00%, and 27.84%, respectively. These results suggest that as the strength of the concrete increases, the brittleness of the concrete increases, and the constraint effect of the steel tube wanes. Although the ultimate displacement of the composite column steadily increases, the ductility coefficient sharply declines, necessitating an increase in the thickness or strength of the steel tube when deploying high-strength concrete in full-scale FUCFST composite columns in practical engineering.

Figure 6c presents load–displacement curves of FUCFSTcs composite columns with varying slenderness ratios. The ultimate axial compressive bearing capacity of the composite column is scarcely affected when the slenderness ratio is less than 3. However, as the slenderness ratio increases, the ultimate bearing capacity of the composite column gradually decreases during the later stages of loading. As the slenderness ratio increases from 2 to 2.25, 2.5, 2.75, and 3, the ultimate axial compressive bearing capacity of the composite column decreases from 104,834 kN to 104,675 kN, 104,467 kN, 104,200 kN, and 103,848 kN, respectively. These reductions equate to 0.15%, 0.35%, 0.60%, and 0.94%, respectively. Conversely, the ultimate displacement for these slenderness ratios increases from 9.50 to 10.81, 12.23, 13.54, and 14.90, respectively. This reflects increases of 13.79%, 28.74%, 42.53%, and 56.84%. However, the ductility factor, decreasing from 1.46 to 1.41, 1.39, 1.37, and 1.36, respectively, decreases by 3.42%, 4.79%, 6.16%, and 6.85%. Although the ultimate axial compressive bearing capacity of the composite column is relatively insensitive to variations in the slenderness ratio within the short column range, as the slenderness ratio increases, the ultimate displacement of the composite column tends to gradually increase, whereas the ductility factor tends to decrease, leading to decreased resistance to deformation energy.

The load–displacement curves of composite columns with different steel tube thicknesses are presented in Figure 6d. It is observed that a significant improvement in the ultimate axial compressive bearing capacity of the composite column is achieved with the increase in steel tube thickness. The load-carrying capacity in the later stage of loading is excellent, and the later-stage bearing capacity gradually increases with the increase in the steel tube thickness. Increases in the ultimate axial compressive bearing capacity from 89,551.5 kN to 93,089.1 kN, 97,551.8 kN, 100,223 kN, 100,661 kN, and 104,467 kN are observed when the steel tube thickness increases from 8 mm to 12 mm, 14 mm, 16 mm, 18 mm, and 20 mm, respectively. This represents increases of 3.95%, 8.93%, 11.92%, 12.41%, and 16.66%, respectively. The ultimate displacement also increases from 11.43 mm to 11.69 mm, 11.81 mm, 11.97 mm, 12.20 mm, and 12.23 mm, respectively, representing increases of 2.97%, 3.32%, 4.72%, 6.74%, and 7.00%, respectively. The ductility factor also increases from 1.23 to 1.33, 1.35, 1.36, 1.37, and 1.39, respectively, representing increases of 8.13%, 9.76%, 10.57%, 11.38%, and 13.01%. Therefore, it is evident that an increase in steel tube thickness leads to improvements in the ultimate axial compressive bearing capacity, ultimate displacement, and ductility factor of FUCFSTcs. This suggests that the constraint effect of the steel tube on the concrete increases, and the brittle failure of UHPC can be slowed down, thereby improving the deformation capacity of the composite column.

The load–displacement curves of composite columns with various cross-sectional sizes are presented in Figure 6e. It is evident that an increase in cross-sectional size significantly improves the ultimate axial compressive bearing capacity of the composite column, as seen in the load–displacement curve, which shows a considerably higher rate of decrease after reaching the peak load. When the circular section radius increases from 300 mm to 350 mm, 400 mm, 450 mm, and 500 mm, the ultimate axial compressive bearing capacity of the composite column increases from 63,098 kN to 91,798.6 kN, 104,467 kN, 129,354 kN, and 155,156 kN, respectively. This represents increases of 29.64%, 65.83%, 105.00%, and 145.90%, respectively. Additionally, the ultimate displacement increases from 9.41 mm to 10.36 mm, 12.63 mm, 13.45 mm, and 14.97 mm, which represent increases of 4.58%, 9.15%, 13.07%, and 15.03%, respectively. However, the ductility coefficient decreases from 1.53 to 1.46, 1.39, 1.33, and 1.30, representing decreases of 10.10%, 29.97%, 42.93%, and 59.09%, respectively. It can be observed that the ultimate axial compressive bearing capacity and ultimate displacement of FUCFSTcs gradually increase with an increase in section size. However, since the restraint effect of the steel tube on concrete progressively decreases with an increase in section size, the combined column resists deformation gradually, and the ductility coefficient decreases accordingly.

## 7. Strength Indicators for FUCFSTcs

The extent to which the cross-sectional load-carrying capacity of FUCFSTcs is affected by the steel tube restraint effect is studied using the strength indicator SI, which is expressed as shown in Equation (4).

(4) $$SI=\frac{N_{u}}{N_{follow}+A_{S}f_{c}}$$

**Figure 7:**
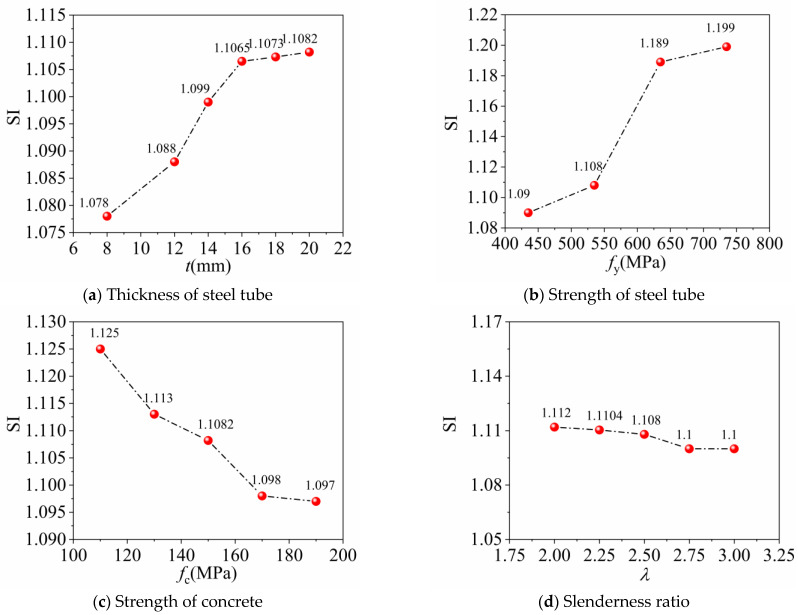
Curves of strength indicators.

The ultimate bearing capacity of FUCFSTcs is derived from a combined column simulation, with *N*_u_ representing this value. The corresponding axial compressive bearing capacity of the hollow steel tube is represented by *N*_follow_. The concrete cross-sectional area is denoted by *A*_s_, while the ultimate value of the compressive strength of the concrete cylinder is represented by *f*_c_. The FUCFSTcs strength index is calculated using Equation (4), with different parameters having the change curves shown in Figure 7. Based on the graphs, it can be observed that the steel tube provides good restraint for UHPC, as the FUCFSTcs strength index remains within the range of 1.078~1.125. The SI value shows a minor decrease only due to the increase in the length-to-slenderness ratio, as can be seen in the graph. This decrease in the ultimate bearing capacity of the combined column is associated with the length-to-slenderness ratio.

## 8. Contribution of Concrete

The concrete contribution ratio (CCR) is an indicator function of the core concrete’s contribution to the overall load-bearing capacity of the combined column and is calculated as shown below:

(5) $$CCR=\frac{N_{u}}{N_{follow}}$$

**Figure 8:**
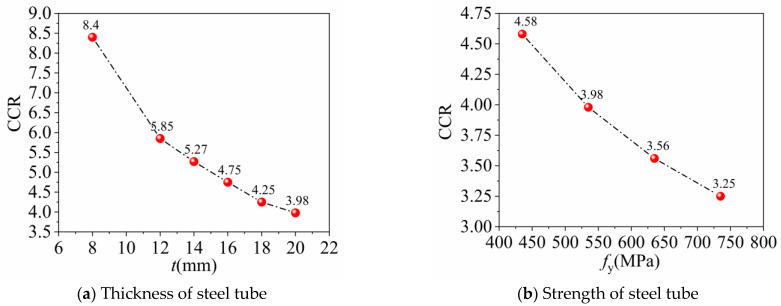
Curves of concrete contribution ratio.

Figure 8 illustrates the change curves of CCR with variation in the steel tube strength and thickness, as calculated by Equation (5). The results demonstrate that the load-bearing effect of concrete on the combined column is noteworthy in cases where the steel tube strength and thickness are low, with the CCR value being more sensitive to the steel tube thickness. With an increase in the steel tube thickness and strength, the restraining effect of the steel tube on the concrete becomes more intense. Consequently, the CCR value is gradually reduced within a small controlled range.

## 9. Analysis of Stress–Strain Curve of Concrete

**Figure 9:**
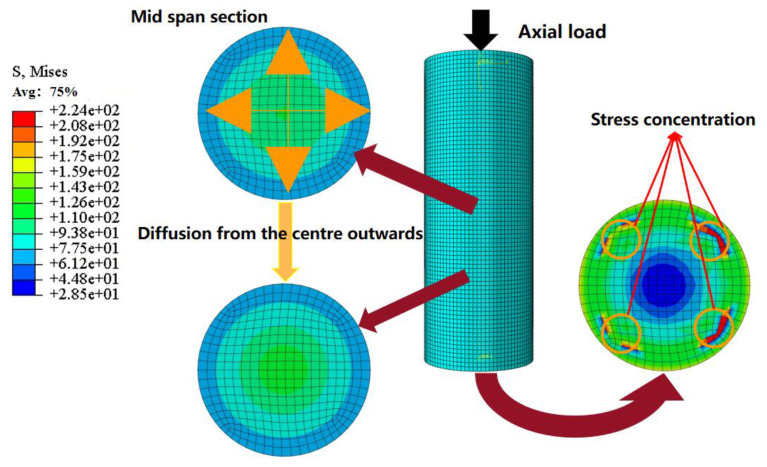
Stress distribution in concrete at different section heights.

**Figure 10:**
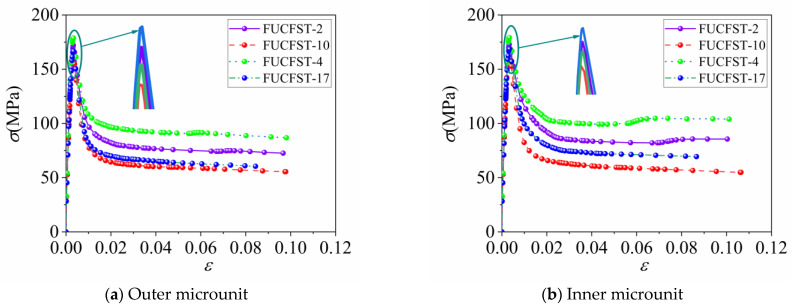
Stress–strain curves of concrete for typical specimens.

To illustrate the stress–strain relationship of concrete in full-scale FUCFST composite columns under axial load, Figure 9 presents the stress distribution at different section heights for a typical specimen FUCFST-2. Additionally, stress–strain curves at two fixed points of the section in columns of typical specimens FUCFST-2, FUCFST-4, FUCFST-10, and FUCFST-17 were selected for comparison, as shown in the curve comparison chart in Figure 10, and the specific parameters of the four groups of specimens are shown in Table 4. The results indicate that the steel tube’s constraint effect on concrete stabilizes the stress–strain curve of concrete in the later stage of loading. The constraint effect of the steel tube on the concrete intensifies with an increase in the steel tube strength and thickness, which leads to an increase in the ultimate stress of the concrete, with the stress significantly increasing in the later stage of loading. As shown in Figure 9, the maximum stress of the concrete in the column section is located in the central area, with the stress value exhibiting a decreasing trend from the center outward. The concrete’s overall stress is relatively uniform and similar to that in the column section at a height of 1500 mm, indicating the uniformity of the concrete’s overall stress distribution. However, stress concentration is observed in the concrete at the end face.

## 10. The Stress Process and Failure Mode of FUCFST Composite Columns

### 10.1. Analysis of the Entire Stress Process of FUCFST Composite Columns

**Figure 11:**
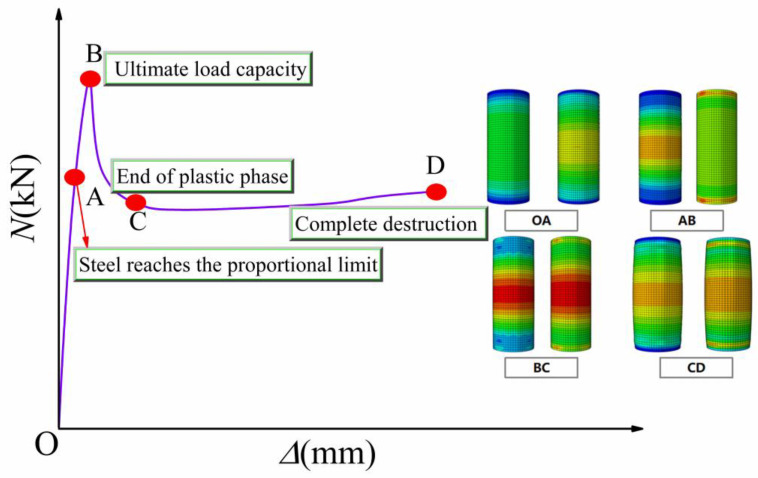
Load–displacement curve and plastic strain cloud map of FUCFESTcs at each stage.

From the load–displacement curve and strain trend of a composite column subjected to an axial compressive load, the stress process was divided into four stages: elastic, plastic, elastic–plastic, and failure stages. The load–displacement curve and strain cloud map for a typical specimen, FUCFST-2, are presented in Figure 11 at each stage.

**Figure 12:**
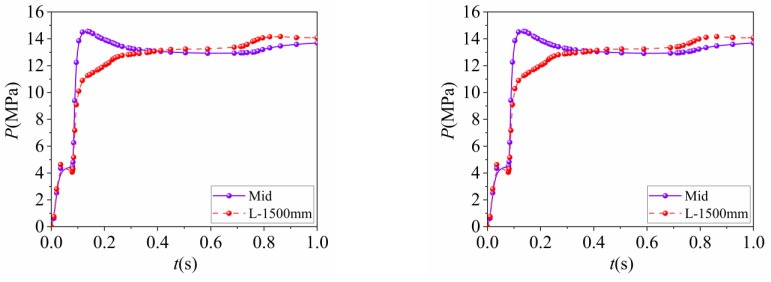
Contact stress between steel tube and concrete.

The elastic stage, which was represented by the OA stage, was where the steel tube and concrete worked independently, resulting in linear load increases with displacement. The contact stress between the steel tube and concrete was small, and the strain distribution of the steel tube and concrete was relatively uniform. As the load increased, the composite column entered the elastic–plastic stage (AB). The load–displacement curve became nonlinear, and the strain of the steel tube was concentrated in the middle of the column, while the concrete experienced stress concentration at its end. In the elastic–plastic stage (BC), as the composite column reached its ultimate axial compressive bearing capacity, the steel tube underwent complete yield, and the strain was concentrated in three regions along the tube, with the maximum in the middle of the column and the minimum at the steel tube end. The extreme value of concrete strain also appeared in the middle of the column, exhibiting a trend of concentration toward the middle of the column. In this stage, the contact stress between the steel tube and concrete increased linearly, indicating the significant outward expansion of the concrete. Subsequently, the composite column entered its failure stage, where the bearing capacity and contact stress between the steel tube and concrete stabilized, and both deformed coordinately, and the contact stress-time curves between steel tubes and concrete are shown in Figure 12. Local buckling of the steel tube at the end became apparent, the concrete in the middle of the column was crushed, and the composite column was ultimately destroyed.

### 10.2. Analysis of FUCFST Composite Column Failure Mode

**Figure 13:**
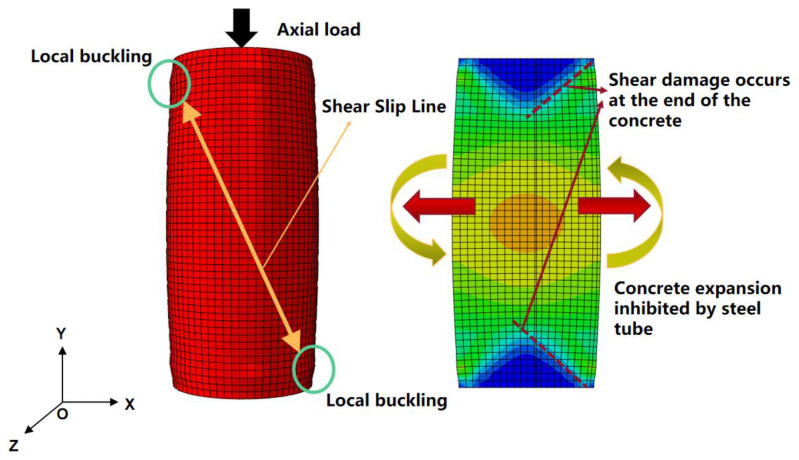
Failure mode of FUCFST-2.

The failure mode of the typical FUCFST-2 specimen is recorded in Figure 13. The composite column mainly exhibits local buckling of the terminus steel tube, buckling of the intermediate steel tube, and shear lines formed at the bulging parts of the upper and lower ends of the steel tube under the action of axial compressive load. The concrete in the middle of the column displays a concentration of strain, and its outward expansion indicates that it has experienced crushing. The strain cloud map of the end concrete exhibits obvious shear slip lines, indicating that it has undergone shear failure.

**Figure 14:**
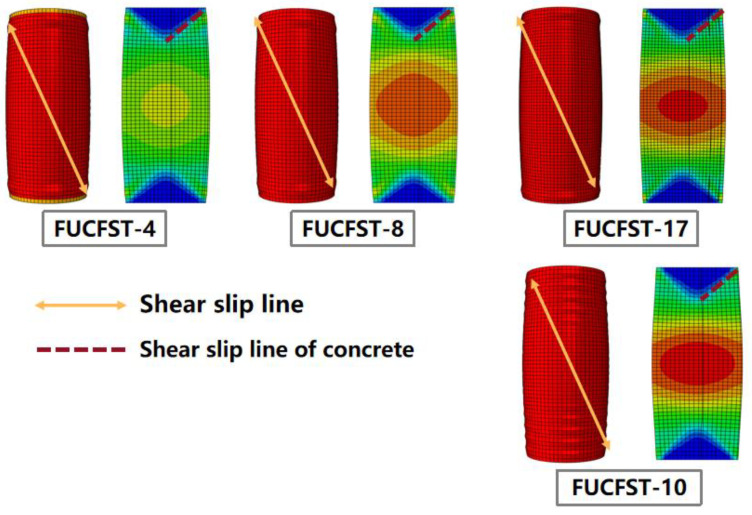
Failure modes of different specimens.

To investigate the failure modes of composite columns under different conditions, we selected four groups of typical specimens, namely, FUCFST-4, FUCFST-8, FUCFST-17, and FUCFST-10, and their failure modes are presented in Figure 14. It is clear that the constraint provided by the steel tube to the concrete increases as the strength of the steel tube increases. This results in the decreased strain of the concrete in the middle of the column. However, for thinner steel tubes with increased section sizes, the constraint of the steel tube on the concrete is reduced, leading to a significant increase in the strain of the concrete in the middle of the column and the maximum strain zone. Therefore, we recommend increasing the strength of the steel tube while decreasing its thickness. Alternately, for increased section sizes, increasing the strength or thickness of the steel tube should be considered.

## 11. Derivation of Ultimate Bearing Capacity Formula for Axial Compression of FUCFSTcs

At present, the main international formulas for calculating the ultimate bearing capacity of concrete-filled steel tubes (CFSTs) are based on the overlay principle proposed by EC4 [24]. The expression of the formulas is shown in Equation (6):

(6) $$N_{\mathrm{EC}4}=A_{S}f_{y}+A_{c}f_{c}$$

**Figure 15:**
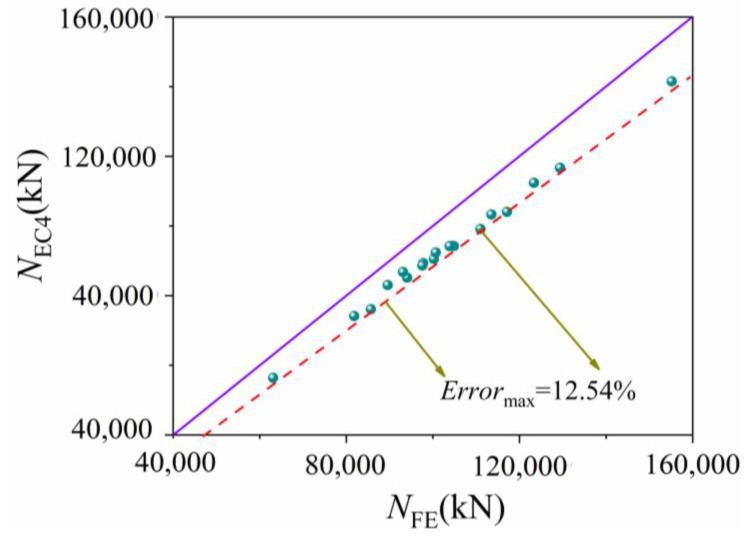
Comparison between EC4 calculation results and finite element results.

Comparing the ultimate bearing capacity of 20 sets of FUCFSTcs calculated using Equation (6) with the simulation results, it can be seen in Figure 15 that the ultimate bearing capacity calculated using Equation (6) is smaller than that calculated using FEM because EC4 does not take into account the constraint effect of the steel tube on the ultimate stress of concrete.

In this paper, the constraint effect coefficient *ξ* is introduced, where *ξ* = (*A*_s_ × *f*_c_)/(*A*_y_ × *f*_y_). Based on this, a formula for calculating the ultimate bearing capacity of full-scale FUCFSTcs is presented, and the constraint effect coefficients of each specimen are shown in Table 4.

(7) $$N_{FT}=A_{S}f_{y}+{\rho A}_{c}f_{c}$$

(8) $$\rho =0.12\xi^{2}-0.106\xi +1.307$$

In the above equations, *A*_s_ is the area of concrete in the cross-section of the composite column, *A*_y_ is the area of the steel tube in the cross-section of the composite column, *f*_y_ is the yield strength of the steel tube, and *f*_c_ is the ultimate value of the compressive strength of the concrete cylinder. The ultimate bearing capacity calculated from Equations (7) and (8) is compared with the results from the finite element analysis in Table 5. The error scatter plot is shown in Figure 15, in which ***N*_FE_** is the ultimate bearing capacity result of the finite element method, ***N*_FT_** is the formula calculation result, the purple solid line represents the error zone of 0, and the upper and lower red dashed lines represent the maximum error zone of 3.04%. It can be seen that the maximum error between the calculated results of the full-scale FUCFCSTcs ultimate bearing capacity formula and the finite element results is only 3.04%, which meets engineering accuracy requirements.

## 12. Conclusions

To explore the axial compression performance of FUCFSTcs, this study established a finite element model using ABAQUS software. By comparing the experimental curves with the simulation results, an extensive parameter analysis was conducted, resulting in the following findings: the load–displacement curve obtained by the finite element method is in good agreement with the experimental curve, with a maximum error of only 6.54% between the ultimate bearing capacity obtained by the finite element method and the experimental value. These findings demonstrate that the UHPC constitutive model adopted in this study can better represent the mechanical properties of UHPC when constrained by steel tubes.

The results of the extended parameter analysis indicate that the ultimate bearing capacity of FUCFSTcs under axial compression increases with the increase in *t*, *f*_y_, *f*_c_, and section size and gradually decreases with the increase in the slenderness ratio.

The slenderness ratio has little effect on the ultimate bearing capacity of short columns under axial compression. This is because the restraining effect of the steel tube on the concrete decreases, leading to a decrease in the resistance to deformation of the composite column. Within the parameter range described in this article, the strength index of FUCFSTcs remains between 1.078 and 1.125, indicating that steel tubes provide a good restraining effect on concrete.

Due to the fact that the calculation formula for concrete-filled steel tubes in EC4 does not take into account the fact that steel tubes can increase the ultimate stress value of concrete by restraining it, its calculation results are all smaller than the finite element results. This article introduces the constraint effect coefficient and statistically regresses the formula for calculating the ultimate bearing capacity of FUCFSTcs under axial compression. The maximum error between the calculation results and the finite element results is 3.04%, which meets engineering accuracy requirements.

## Figures and Tables

**Table 1:** Specific parameters of 21 FUCFSTcs test pieces.

Specimens	*f*c/MPa	*f*y/MPa	*D*/mm	*t*/mm	*L*/mm	*λ*
FUCFST-1	150	435	800	20	2000	2.5
FUCFST-2	150	535	800	20	2000	2.5
FUCFST-3	150	635	800	20	2000	2.5
FUCFST-4	150	735	800	20	2000	2.5
FUCFST-5	110	535	800	20	2000	2.5
FUCFST-6	130	535	800	20	2000	2.5
FUCFST-7	170	535	800	20	2000	2.5
FUCFST-8	190	535	800	20	2000	2.5
FUCFST-9	150	535	800	8	2000	2.5
FUCFST-10	150	535	800	12	2000	2.5
FUCFST-11	150	535	800	14	2000	2.5
FUCFST-12	150	535	800	16	2000	2.5
FUCFST-13	150	535	800	18	2000	2.5
FUCFST-14	150	535	600	20	1500	2.5
FUCFST-15	150	535	700	20	1750	2.5
FUCFST-16	150	535	900	20	2250	2.5
FUCFST-17	150	535	1000	20	2500	2.5
FUCFST-18	150	535	800	20	1600	2
FUCFST-19	150	535	800	20	1800	2.25
FUCFST-20	150	535	800	20	2200	2.75
FUCFST-21	150	535	800	20	2400	3

**Table 2:** Specific parameters of experimental composite columns.

Specimens		*D*/mm	*t*/mm	*f*_c_ (MPa)	*f*_y_ (MPa)	*L*/mm	*N*_t_/kN	*N*_a_/kN	Error
Chen [10]	CSC1-2	113.7	2.06	113.2	269.9	342	1487	1589.10	6.54%
	CSC1-3	113.7	2.05	130.8	269.9	342	1535	1622.35	5.38%
	CSC2-2	114.9	4.01	113.2	304.3	342	1631	1598.23	2.05%
	CSC2-3	114.9	4.01	130.8	304.3	342	1748	1741.07	0.4%
	CSC3-2	107.9	8.01	113.2	251.8	342	1482	1522.17	2.64%
	CSC3-3	107.9	8.03	130.8	251.8	342	1613	1632.87	1.22%
Richard [23]	S1-3-1a	114	3.6	173	406	250	2399	2432	1.36%
	S1-3-1b	114	3.6	173	406	250	2399	2432	1.36%
Wei [15]	UCS-2	140	6.2	142.1	1153	420	5582.38	5386.75	3.71%
	UCS-3	140	10.4	142.1	773	420	6354.45	6339.26	0.53%
	UCS-4	140	8.3	142.1	813	420	5310.30	5502.35	3.88%
	UCS-5	140	6.2	142.1	359	420	3186.18	3202.47	4.73%

Note: *N*_t_ is the test ultimate bearing capacity, and *N*_a_ is the finite element ultimate bearing capacity.

**Table 3:** Mechanical properties of 20 full-scale FUCFST composite columns.

Specimens	*f*y/MPa	*N*_a_/kN	∆_m_/mm	∆_y_/mm	∆_u_/mm	*μ*
FUCFST-1	435	97,758.9	11.96	9.95	13.43	1.35
FUCFST-2	535	104,467	12.23	9.90	13.76	1.39
FUCFST-3	635	110,960	12.45	9.95	14.03	1.41
FUCFST-4	735	117,093	12.63	10.01	14.31	1.43
FUCFST-5	535	85,665.4	11.07	8.44	14.85	1.76
FUCFST-6	535	94,037.9	11.59	9.54	14.12	1.48
FUCFST-7	535	113,527	12.54	10.51	13.87	1.32
FUCFST-8	535	123,351	13.37	11.22	14.25	1.27
FUCFST-9	535	89,551.5	11.43	10.09	12.41	1.23
FUCFST-10	535	93,089.1	11.09	9.73	12.94	1.33
FUCFST-11	535	97,551.8	11.81	10.02	13.53	1.35
FUCFST-12	535	100,223	11.97	10.05	13.67	1.36
FUCFST-13	535	100,661	12.20	9.73	13.33	1.37
FUCFST-14	535	63,098	9.41	7.31	11.18	1.53
FUCFST-15	535	81,798.6	10.36	8.50	12.41	1.46
FUCFST-16	535	129,354	13.45	11.23	14.94	1.33
FUCFST-17	535	155,156	14.97	12.43	16.16	1.3
FUCFST-18	535	104,834	9.5	7.76	11.33	1.46
FUCFST-19	535	104,675	10.81	8.92	12.40	1.39
FUCFST-20	535	104,200	13.54	10.96	15.02	1.37
FUCFST-21	535	103,848	14.9	11.87	16.50	1.39

Note: In the table, ∆_m_ is the ultimate displacement, ∆_y_ is the yield displacement, and ∆_u_ is the corresponding displacement when the bearing capacity decreases to 80%.

**Table 4:** Specific parameters of 4 sets of typical specimens.

Specimens	*f*c/MPa	*f*y/MPa	*D*/mm	*t*/mm	*L*/mm	*λ*
FUCFST-2	150	535	800	20	2000	2.5
FUCFST-4	150	735	800	20	2000	2.5
FUCFST-10	150	535	800	12	2000	2.5
FUCFST-17	150	535	1000	20	2500	2.5

**Table 5:** Comparing finite element results with calculated results.

Specimens	(*A*_s_ × *f*_c_)/1000	(*A*_y_ × *f*_y_)/1000	*ξ*	*N*_FE_/kN	*N*_FT_/kN	|(*N*_FE_ − *N*_FT_)/*N*_FE_|
FUCFST-1	68,046.90	21,318.85	1.82	97,758.9	99,965.29	2.26%
FUCFST-2	68,046.90	26,219.73	1.92	104,467	104,470.18	0.00%
FUCFST-3	68,046.90	31,120.62	2.02	110,960	108,992.57	1.77%
FUCFST-4	68,046.90	36,021.50	2.12	117,093	113,532.48	3.04%
FUCFST-5	49,901.06	26,219.73	1.55	85,665.4	84,742.84	1.08%
FUCFST-6	58,973.98	26,219.73	1.74	94,037.9	94,684.10	0.69%
FUCFST-7	77,119.82	26,219.73	2.11	113,527	114,125.09	0.53%
FUCFST-8	86,192.73	26,219.73	2.29	123,351	123,672.84	0.26%
FUCFST-9	72,412.45	10,649.24	4.17	89,551.5	89,363.93	0.21%
FUCFST-10	70,942.19	15,893.19	2.92	93,089.1	94,349.39	1.35%
FUCFST-11	70,212.71	18,495.00	2.57	97,551.8	97,041.48	0.52%
FUCFST-12	69,487.00	21,083.35	2.30	100,223	99,633.31	0.59%
FUCFST-13	68,765.06	23,658.27	2.09	100,661	102,107.28	1.44%
FUCFST-14	36,945.13	19,496.72	1.55	63,098	62,836.18	0.41%
FUCFST-15	51,317.92	22,858.23	1.74	81,798.6	82,441.54	0.79%
FUCFST-16	87,132.07	29,581.24	2.11	129,354	128,888.83	0.36%
FUCFST-17	108,573.44	32,942.74	2.30	155,156	155,677	0.34%
FUCFST-18	68,046.90	26,219.73	1.92	104,834	104,470	0.35%
FUCFST-19	68,046.90	26,219.73	1.92	104,675	104,470	0.20%
FUCFST-20	68,046.90	26,219.73	1.92	104,200	104,470	0.26%
FUCFST-21	68,046.90	26,219.73	1.92	103,848	104,470	0.60%

## Data Availability

The raw data supporting the conclusion of this article will be made available by the authors without undue reservation.
